# A Rare Suprasellar Ependymoma With Negative Targeted Fusion Testing: Diagnostic Value of Histopathological, Immunohistochemical, and Ultrastructural Findings

**DOI:** 10.7759/cureus.117336

**Published:** 2026-10-01

**Authors:** Ren Fujiwara, Toshio Hirohata, Kenichi Oyama, Keisuke Onoda, Akira Matsuno

**Affiliations:** 1 Department of Neurosurgery, Mejiro Daini Hospital, Tokyo, JPN; 2 Department of Neurosurgery, Hongo Neurosurgery Clinic, Saitama, JPN; 3 Department of Neurosurgery, International University of Health and Welfare Mita Hospital, Tokyo, JPN; 4 Department of Neurosurgery, International University of Health and Welfare Narita Hospital, Narita, JPN

**Keywords:** ependymoma, gene fusion, pituitary mass, suprasellar tumor, transsphenoidal surgery

## Abstract

Ependymomas arising in the supratentorial region preferentially appear in the third ventricle or cerebral parenchyma, making a suprasellar location extremely rare. We report a case of a 36-year-old woman who presented with incomplete bitemporal hemianopsia and was found to have a suprasellar mass lesion on magnetic resonance imaging. The patient underwent endoscopic transsphenoidal surgery with gross total resection. Pathological analysis of the resected tumor demonstrated perivascular pseudorosettes and immunohistochemical positivity for glial fibrillary acidic protein, vimentin, cytokeratin CAM5.2, and epithelial membrane antigen. Ultrastructural findings revealed intercellular junctional complexes, microvilli, and intermediate filaments on electron microscopy. Molecular analysis using reverse transcription-polymerase chain reaction failed to detect a gene fusion. According to the WHO classification 2021, the tumor was diagnosed as supratentorial ependymoma NEC (not elsewhere classified). To our knowledge, only a small number of cases of ependymoma developing in the suprasellar region have been reported in the literature. Radial glia-like cells present in the adult central nervous system, specifically in the subventricular zone, may serve as the origin for adult supratentorial ependymomas. This case highlights that suprasellar ependymoma, though rare, should be considered in the differential diagnosis of sellar-suprasellar tumors. Molecular testing for defining gene fusions is important for precise classification of future cases.

## Introduction

The suprasellar region presents unique diagnostic challenges due to the variety of pathological entities that can occur in this anatomical location. Pituitary adenomas represent the most common suprasellar masses, accounting for approximately 10%-15% of all intracranial tumors [1]. These benign neoplasms arise from adenohypophyseal cells and can present with endocrinological dysfunction, mass effect symptoms, or both [1]. Craniopharyngiomas, the second most frequent suprasellar lesions, arise from embryonic remnants of Rathke's pouch and demonstrate characteristic calcifications and cyst formation on neuroimaging [1]. Other differential diagnoses include Rathke's cleft cysts, which are benign epithelial-lined cysts arising from incomplete regression of Rathke's pouch, meningiomas originating from the tuberculum sellae or diaphragma sellae, germ cell tumors, and metastatic lesions. Each of these entities presents distinct radiological, clinical, and histopathological features that guide appropriate therapeutic management [1].

Ependymomas are glial neoplasms that demonstrate ependymal differentiation and primarily arise in regions where ependymal cells are naturally present [2]. These tumors account for approximately 2%-3% of all intracranial neoplasms and show a bimodal age distribution [2,3]. In the pediatric population, ependymomas predominantly occur in the posterior fossa, particularly within the fourth ventricle. Adult ependymomas more commonly involve the spinal cord, where they constitute the most frequent intramedullary tumor [4]. When ependymomas do occur in the supratentorial compartment, they typically arise in the lateral ventricles, third ventricle, or within the cerebral parenchyma, often maintaining some connection to the ventricular system [2,5,6].

The molecular landscape of ependymomas has been revolutionized by recent advances in genomic profiling and next-generation sequencing technologies. The 2021 World Health Organization Classification of Tumors of the Central Nervous System has incorporated molecular features as essential diagnostic criteria [7]. Supratentorial ependymomas are now defined by specific gene fusions, and two molecularly defined types are recognized: ZFTA fusion-positive and YAP1 fusion-positive ependymomas [7]. ZFTA fusion-positive tumors are the more common of the two, whereas YAP1 fusion-positive ependymomas are considerably less frequent and occur predominantly in young children [7].

The occurrence of ependymomas in the sellar and suprasellar regions represents an exceptionally rare phenomenon, with only a small number of cases documented in the worldwide medical literature over the past seven decades [8,9]. The most widely accepted hypothesis suggests an origin from ectopic ependymal cell rests derived from the embryonic ependymal cleft, a transient structure that forms part of the developing pituitary infundibulum [10,11].

## Case presentation

Clinical presentation

A 36-year-old woman presented to our institution with a chief complaint of progressive visual field defects that had been worsening over the course of 15 months. The patient had initially sought medical attention at a local hospital, where a suprasellar mass was identified on magnetic resonance imaging. Her visual symptoms were characterized by incomplete bitemporal hemianopsia, which showed gradual deterioration. The patient reported that this deterioration was more noticeable during menstrual periods, although a hormonal influence on tumor growth or peritumoral edema was not established. The patient's medical history was significant for menstrual irregularities managed with oral contraceptive pills for several years. She denied any history of severe headaches, galactorrhea, cold intolerance, fatigue, or other symptoms suggestive of pituitary dysfunction. Family history was unremarkable for endocrine disorders, intracranial neoplasms, or hereditary cancer syndromes. Physical examination revealed no focal neurological deficits apart from the visual field abnormalities, and there were no signs of acromegaly or Cushing's syndrome. Comprehensive ophthalmological evaluation confirmed incomplete bitemporal hemianopsia, consistent with optic chiasmal compression from a suprasellar mass. Visual acuity was preserved bilaterally, and fundoscopic examination revealed no evidence of papilledema or optic atrophy. Endocrinological assessment revealed an elevated serum prolactin level of 333 ng/mL, suggesting either a prolactin-secreting tumor or stalk compression effect. Provocative testing of anterior pituitary hormone function demonstrated hypogonadotropic hypogonadism with suppressed luteinizing hormone and follicle-stimulating hormone levels. Growth hormone stimulation testing using growth hormone-releasing peptide-2 revealed severe adult growth hormone deficiency, with a peak growth hormone concentration of 4.0 ng/mL, well below the normal response threshold. Insulin-like growth factor-1 levels were significantly reduced, with a standard deviation score of -3.2 based on age- and sex-adjusted reference ranges established for the Japanese population [12]. The pre- and postoperative endocrinological findings are summarized in Table 1.

**Table 1:** Pre- and postoperative endocrinological findings GH values shown in the table represent basal levels. Peak GH concentrations during the GHRP-2 stimulation test were 4.0 ng/mL preoperatively and 2.9 ng/mL postoperatively. LH, luteinizing hormone; FSH, follicle-stimulating hormone; ACTH, adrenocorticotropic hormone; GH, growth hormone; IGF-1, insulin-like growth factor 1; SD, standard deviation; TSH, thyroid-stimulating hormone; T4, thyroxine; T3, triiodothyronine

Parameter	Normal range	Preoperatively	3 months postoperatively
LH (mIU/mL)	2.4-12.6 (follicular)	<0.10	<0.10
FSH (mIU/mL)	3.5-12.5 (follicular)	0.34	1.27
Estradiol (pg/mL)	12.5-166 (follicular)	11	10
ACTH (pg/mL)	7.2-63.3	22.7	3.9
Cortisol (μg/dL)	6.2-19.4	17.5	5.9
Prolactin (ng/mL)	4.8-23.3	333	122
GH (ng/mL)	-	0.82	0.95
IGF-1 (ng/mL)	Age-adjusted	80 (SD score -3.2)	68 (SD score -3.8)
TSH (μIU/mL)	0.5-5.0	2.1	1.05
Free T4 (ng/dL)	0.93-1.70	0.94	0.64
Free T3 (ng/dL)	2.30-4.30	2.46	2.29

Magnetic resonance imaging of the brain revealed a well-circumscribed, round suprasellar mass measuring approximately 20 mm in diameter, with heterogeneous enhancement following gadolinium administration (Figure 1). The lesion demonstrated compression of the optic chiasm with superior displacement, explaining the patient's visual symptoms. Notably, the sella turcica appeared normal in size, and the normal pituitary gland was clearly identifiable within the sellar compartment, indicating a purely suprasellar origin of the mass. The imaging characteristics showed intermediate signal intensity on T1-weighted images and slightly hyperintense signal on T2-weighted sequences. The enhancement pattern was heterogeneous but predominantly solid, with no evidence of calcification or cystic components. These findings were consistent with a suprasellar mass, with the differential diagnosis including pituitary adenoma with suprasellar extension, craniopharyngioma, pituicytoma, Rathke's cleft cyst, meningioma, and, rarely, other neoplastic processes.

**Figure 1:**
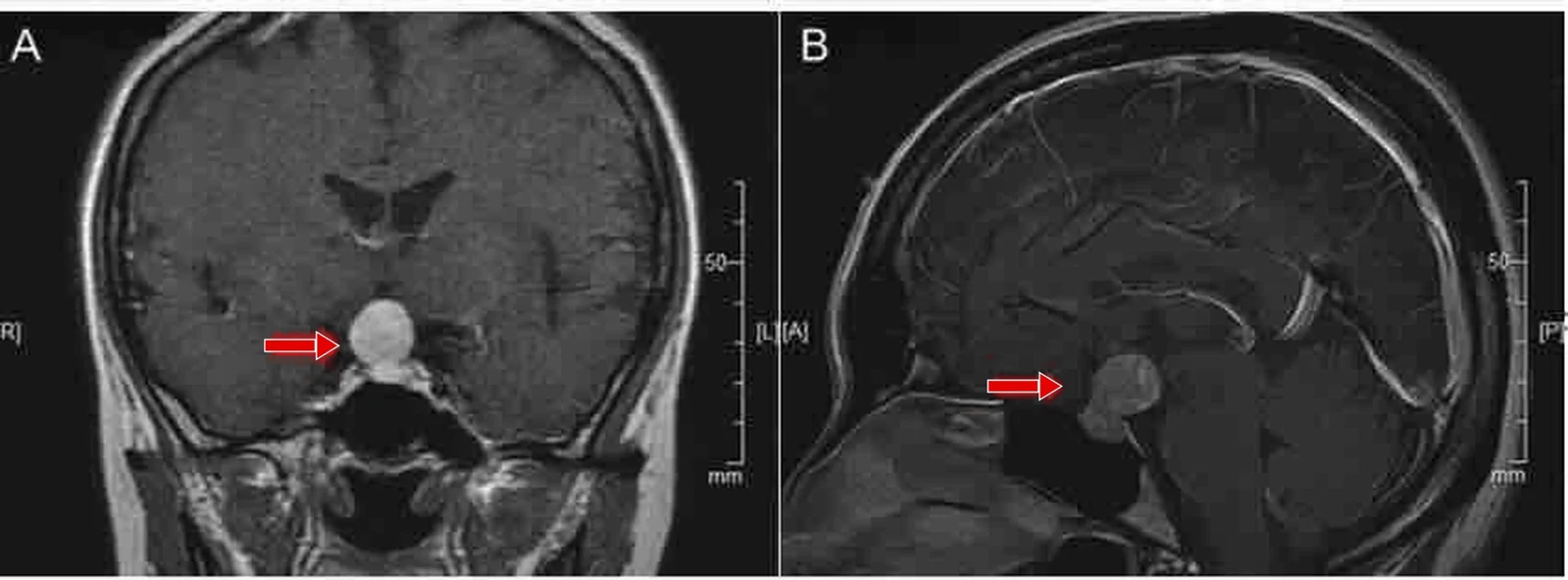
Preoperative MRI with gadolinium enhancement showing a suprasellar tumor: (A) coronal and (B) sagittal sections The well-circumscribed, heterogeneously enhancing mass (arrows) compresses the optic chiasm superiorly. Note the normal-sized sella turcica and preserved normal pituitary gland within the sellar compartment, confirming the purely suprasellar location of the lesion.

Given the progressive visual deterioration and evidence of pituitary dysfunction, surgical intervention was recommended after multidisciplinary discussion involving the neurosurgery, endocrinology, and ophthalmology teams. The patient provided informed consent for endoscopic transsphenoidal surgery under general anesthesia. Intraoperatively, the normal pituitary gland was found to completely occupy the intrasellar space, confirming the purely suprasellar location of the mass. The tumor appeared well vascularized, firm, and rubbery in consistency, which was atypical for a pituitary adenoma and raised suspicion for an alternative diagnosis. The lesion was located rostral to the sellar diaphragm and demonstrated no direct connection to the pituitary gland. The tumor was successfully removed through careful piecemeal resection, achieving gross total resection as confirmed by intraoperative visualization and postoperative imaging (Figure 2). During the procedure, the floor of the third ventricle was inadvertently opened, revealing the intimate relationship between the tumor and the hypothalamo-neurohypophyseal axis.

**Figure 2:**
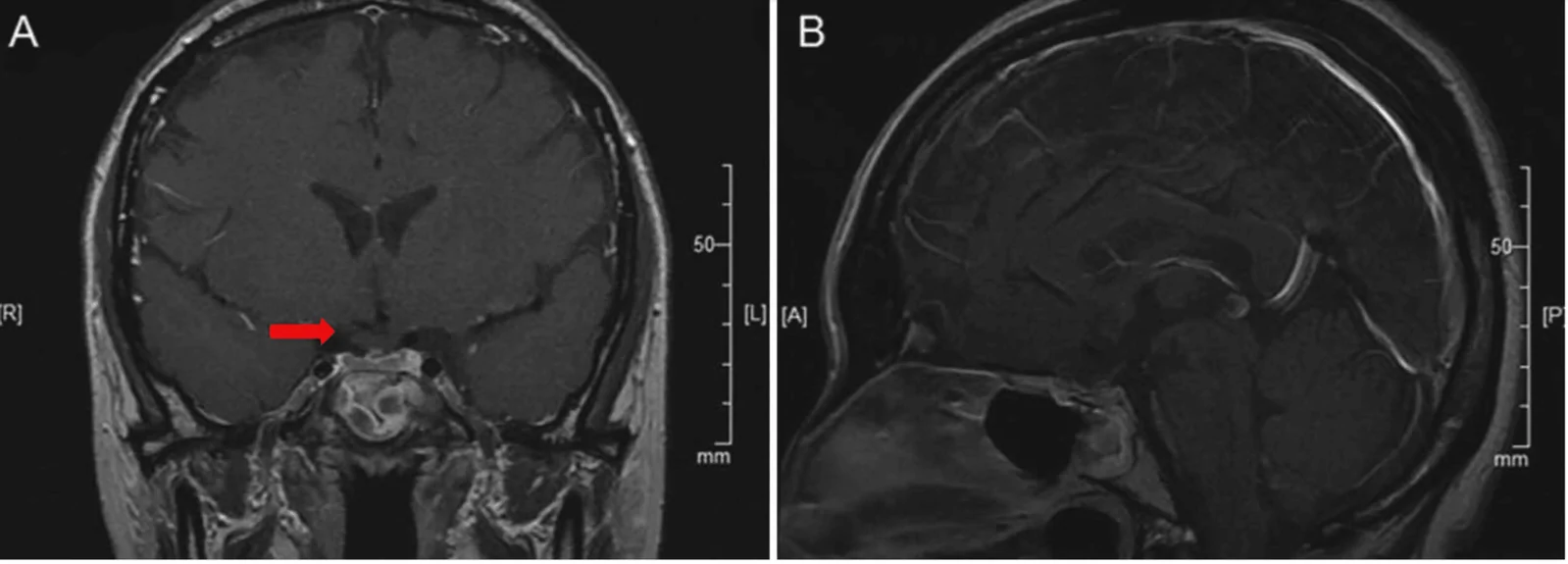
Postoperative MRI with gadolinium enhancement: (A) coronal and (B) sagittal sections Complete tumor resection is demonstrated, with resolution of optic chiasm compression (arrows indicate the decompressed chiasm). The normal pituitary gland remains intact within the sella turcica, and no residual enhancing tumor tissue is visible in the suprasellar region.

Postoperatively, the patient developed transient central diabetes insipidus, a not uncommon complication following surgery in the suprasellar region, which was successfully managed with intranasal desmopressin administration. The diabetes insipidus gradually resolved over several weeks, allowing discontinuation of hormone replacement. Three months following surgery, serum prolactin levels remained elevated at 122 ng/mL, prompting initiation of cabergoline therapy at 0.5 mg twice weekly. This dopamine agonist treatment successfully normalized prolactin levels to 8 ng/mL by the six-month follow-up visit. The patient's hypogonadotropic hypogonadism persisted, necessitating hormone replacement therapy with estrogen and progesterone using the Kaufmann treatment protocol to restore menstrual cycles and prevent long-term complications of estrogen deficiency.

Pathological findings

The resected tissue specimens were processed according to standard neuropathological protocols established by our institution. Tissues were fixed in 10% neutral-buffered formalin for 24 hours to ensure adequate penetration and preservation of morphological details, then dehydrated through graded alcohols, cleared in xylene, and embedded in paraffin blocks. Serial sections were cut at 4-μm thickness using a rotary microtome and mounted on positively charged glass slides for routine hematoxylin and eosin staining and immunohistochemical analysis. Immunohistochemical staining was performed using an automated Ventana Benchmark Ultra system (Ventana Medical Systems, Tucson, Arizona, USA) following the manufacturer's protocols. A comprehensive panel of commercially available primary antibodies was employed, including prolactin (ICN, Costa Mesa, CA, USA; 1:100), luteinizing hormone (DAKO, Carpinteria, CA, USA; M3502, 1:100), follicle-stimulating hormone (DAKO; M3504, 1:200), growth hormone (DAKO; M1561, 1:7), adrenocorticotropic hormone (DAKO; M3507, 1:600), thyroid-stimulating hormone (DAKO; M3503, 1:200), glial fibrillary acidic protein (GFAP) (DAKO; M0761, 1:100), vimentin (DAKO; M0725, 1:200), CD31 (Novocastra, Burlingame, CA, USA; NCL-1A10, 1:50), cytokeratin AE1/AE3 (DAKO; M3515, 1:100), cytokeratin CAM5.2 (Becton Dickinson, San Jose, CA, USA; 439205, prediluted), epithelial membrane antigen (EMA) (DAKO; M0613, 1:100), CD99 (Novocastra; NCL-CD99, 1:100), and Ki-67 (Invitrogen, Carlsbad, CA, USA; ZYMED, prediluted).

Microscopic examination revealed tumor tissue with characteristic pseudopapillary architecture composed of neoplastic cells arranged around central blood vessels. The tumor cells displayed oval to elongated nuclei with fine chromatin and moderate amounts of eosinophilic cytoplasm containing prominent rough endoplasmic reticulum. The most distinctive and diagnostically significant feature was the presence of perivascular pseudorosettes, in which tumor cells were arranged radially around blood vessels with intervening anuclear zones containing delicate fibrillary processes (Figure 3A, 3B). These structures are a characteristic histological feature of ependymal differentiation and represent the cellular arrangement around blood vessels, with tumor cell processes extending toward the vascular wall. The stromal component contained abundant well-formed blood vessels lined by endothelial cells, demonstrating characteristic morphology consistent with the perivascular pattern of tumor organization.

**Figure 3:**
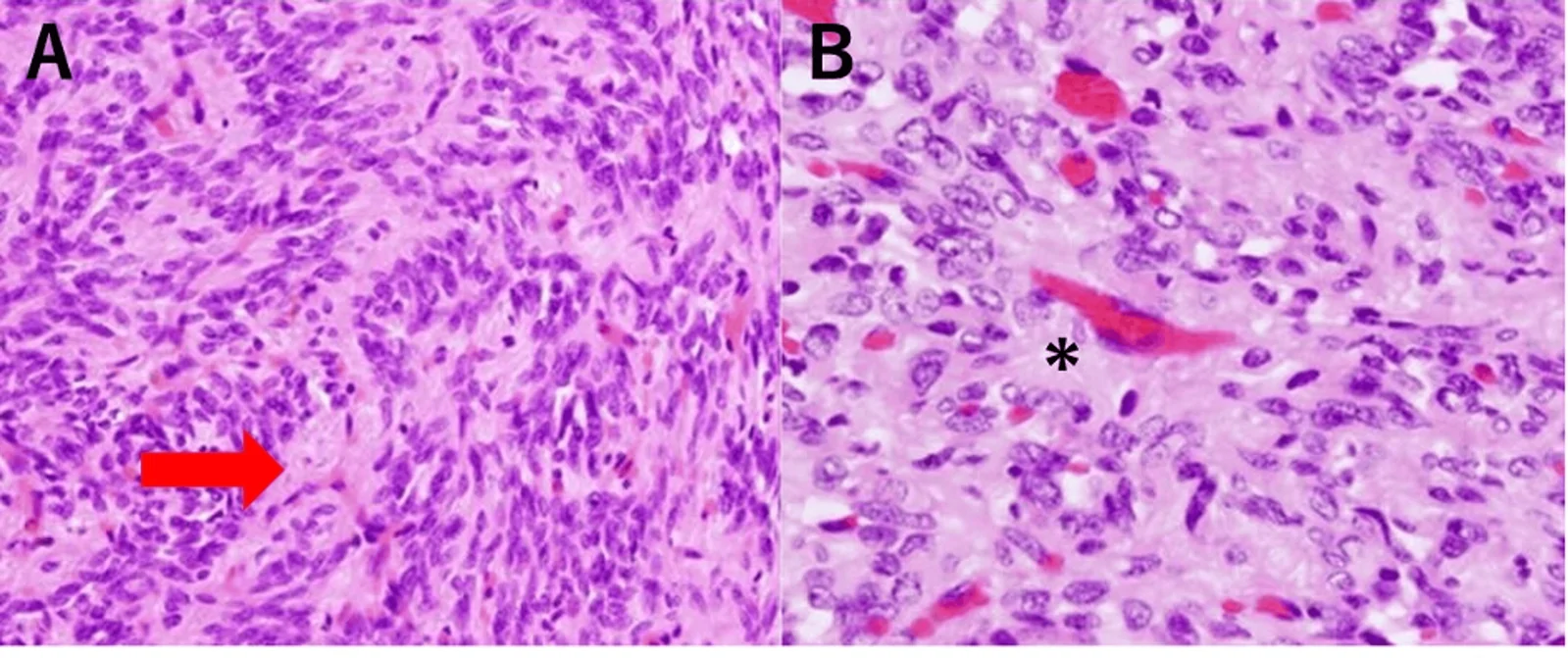
Pathological findings of the resected specimens. (A) Pseudopapillary architecture with abundant stromal microvessels (hematoxylin-eosin stain, original magnification ×20). (B) Perivascular pseudorosettes with tumor cells displaying oval nuclei and eosinophilic endoplasmic reticulum (hematoxylin-eosin stain, original magnification ×40) Note the characteristic perivascular arrangement of tumor cells around central blood vessels (arrow in A). The anuclear zones (asterisk in B) between tumor cells and blood vessels are characteristic of ependymal differentiation, with tumor cell processes extending radially toward the vascular wall.

Immunohistochemical analysis revealed a pattern highly characteristic of ependymal differentiation. The tumor cells demonstrated strong and diffuse positivity for GFAP, indicating glial lineage (Figure 4A). The tumor cells demonstrated diffuse vimentin expression, a finding frequently encountered in ependymal tumors (Figure 4B). Focal CAM5.2 expression was identified, consistent with the known expression of epithelial-associated markers in a subset of ependymomas (Figure 4C). The EMA staining pattern showed characteristic punctate, dot-like cytoplasmic positivity, a highly characteristic immunohistochemical finding in ependymomas (Figure 4D). This pattern results from immunoreactivity of intercellular microlumina and represents one of the most reliable immunohistochemical markers for ependymal differentiation. Immunohistochemistry for isocitrate dehydrogenase 1 (IDH1) R132H and thyroid transcription factor 1 (TTF-1) was negative, findings that argue against IDH-mutant glioma and metastatic carcinoma of pulmonary or thyroid origin, respectively. CD99 and CD34 were not expressed in tumor cells. The Ki-67 proliferation index was low, with less than 1% of tumor cells showing nuclear positivity, indicating a low proliferative index. All anterior pituitary hormones, including prolactin, luteinizing hormone, follicle-stimulating hormone, growth hormone, adrenocorticotropic hormone, and thyroid-stimulating hormone, were immunonegative, confirming that the tumor did not arise from pituitary tissue.

**Figure 4:**
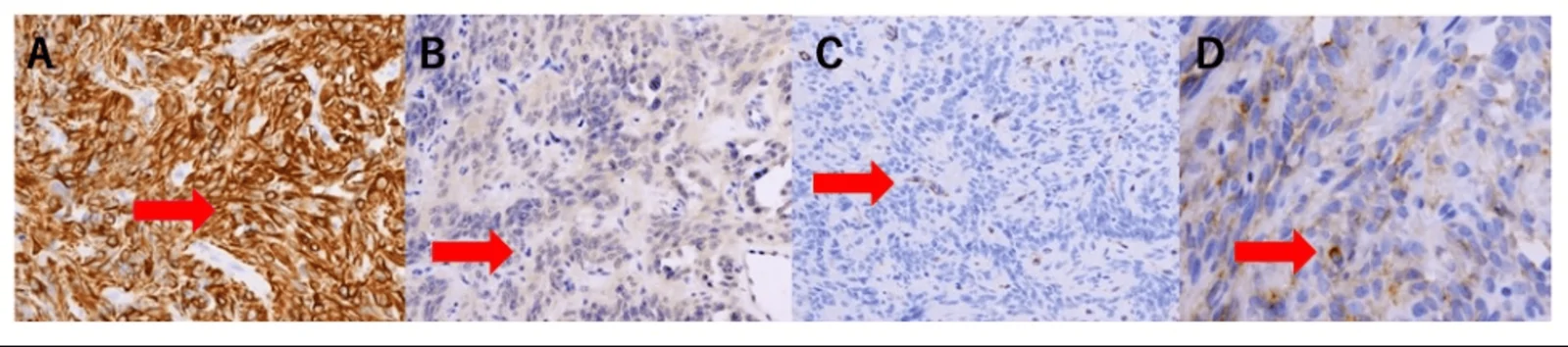
Immunohistochemical staining demonstrating ependymal differentiation (A) Strong and diffuse positivity for GFAP (original magnification ×40), indicating glial lineage, with prominent cytoplasmic staining throughout the tumor cells (arrows). (B) Diffuse positivity for vimentin (original magnification ×40), a finding frequently encountered in ependymal tumors, with intense cytoplasmic reactivity (arrows). (C) Focal positivity for cytokeratin CAM5.2 (original magnification ×20), consistent with the expression of epithelial-associated markers in a subset of ependymomas (arrows indicate positive cells). (D) EMA showing characteristic punctate, dot-like cytoplasmic positivity (original magnification ×40). The distinctive dot-like pattern (arrows) is highly characteristic of ependymomas and represents immunoreactivity of intercellular microlumina. GFAP, glial fibrillary acidic protein; EMA, epithelial membrane antigen

Ultrastructural examination was performed on representative tissue samples that had been fixed in 2.5% glutaraldehyde in phosphate buffer, post-fixed in 1% osmium tetroxide, dehydrated through graded alcohols, and embedded in epoxy resin. Ultrathin sections were cut using an ultramicrotome, stained with uranyl acetate and lead citrate, and examined under a JEM-1200EX transmission electron microscope (JEOL, Tokyo, Japan) operated at 80 kV. Electron microscopy revealed ultrastructural features that provided supportive evidence of ependymal differentiation. These features included intercellular junctional complexes between adjacent tumor cells, intercellular microlumina lined by microvilli, and abundant intermediate filaments within cellular processes (Figure 5A, 5B, 5C). The intercellular junctions demonstrated the typical electron-dense plaques characteristic of ependymal cells, while the microlumina contained variable numbers of microvilli projecting into the luminal space. The cytoplasm contained well-developed organelles, including rough endoplasmic reticulum, Golgi apparatus, and mitochondria, reflecting active protein synthesis typical of differentiated ependymal cells.

**Figure 5:**
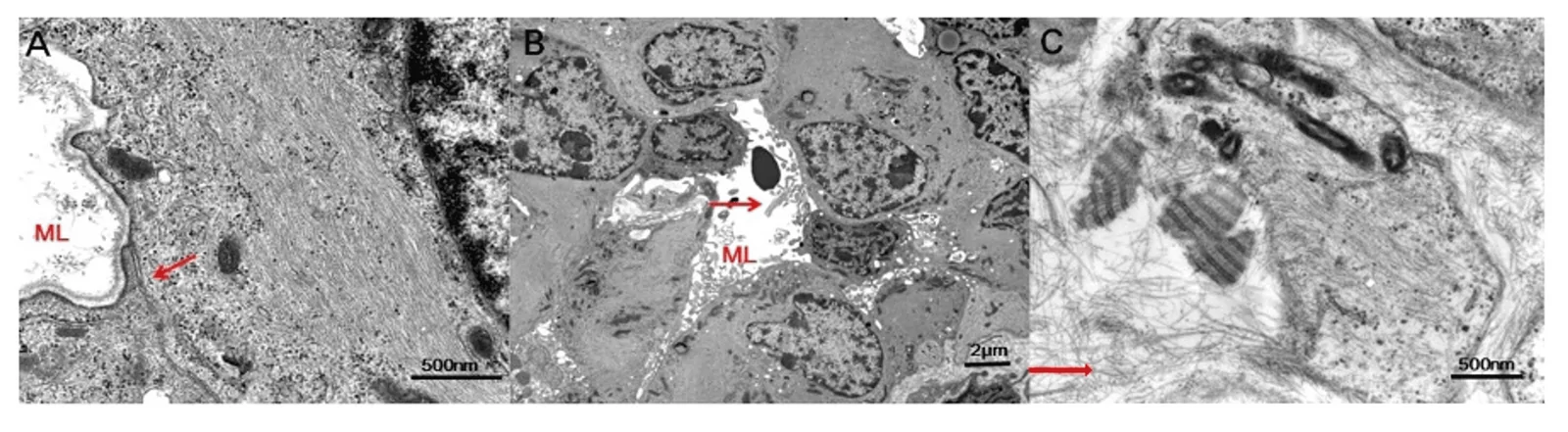
Ultrastructural findings supporting ependymal differentiation (A) Intercellular junctional complex (arrow) between adjacent tumor cells with an intercellular ML (original magnification ×25,000). (B) Intercellular ML containing multiple microvilli (arrows) projecting into the luminal space (original magnification ×2,500). (C) Abundant intermediate filaments within cellular processes (arrows) (original magnification ×15,000). The electron-dense junctional complex in (A) is characteristic of ependymal cells and maintains cellular adhesion. These microvilli-lined microlumina in (B) are characteristic ultrastructural features of ependymal differentiation. The intermediate filaments in (C) demonstrate the cytoskeletal organization typical of differentiated ependymal cells and contribute to cellular structural integrity. ML, microlumen

Molecular analysis was performed using reverse transcription-polymerase chain reaction (RT-PCR) following RNA extraction from formalin-fixed, paraffin-embedded tissue sections. The targeted assay employed primers designed to detect ZFTA (formerly C11orf95)/RELA fusion, YAP1 fusions, including YAP1-MAMLD1 and YAP1-FAM118B, and MAML2 fusion. None of these fusion transcripts was detected. Because this was a targeted RT-PCR assay performed on formalin-fixed, paraffin-embedded material, RNA degradation may lead to false-negative results, and fusions outside the assay panel would not be detected. Methylation profiling was not performed. The negative result therefore does not exclude the diagnosis of ependymoma, as a subset of cases lacks currently identifiable gene fusions. Based on the combination of characteristic histomorphological features, specific immunohistochemical profile, diagnostic ultrastructural characteristics, and clinical presentation, the tumor was classified as supratentorial ependymoma, NEC (not elsewhere classified), according to the 2021 WHO Classification of Tumors of the Central Nervous System.

## Discussion

Ependymomas are glial tumors characterized by ependymal differentiation. In children, they commonly arise in the posterior fossa, particularly within the fourth ventricle, while in adults, they predominantly involve the spinal cord and posterior fossa. Supratentorial ependymomas frequently develop in the lateral or third ventricles or intraparenchymally [6]. The sellar region, however, is an exceptionally rare site for ependymoma development. Only a small number of sellar and suprasellar ependymomas have been reported in the literature [8-11,13-23]. Selected previously reported cases and their clinicopathological characteristics are summarized in Table 2. Molecular analyses were not reported in most of these previous reports.

**Table 2:** Selected previously reported sellar and suprasellar ependymomas M, male; F, female; GFAP, glial fibrillary acidic protein; EMA, epithelial membrane antigen

Year/reference	Authors	Age	Sex	Treatment	Pathology
1989 [10]	Winer et al.	81	M	Transsphenoidal, partial resection	Typical histology of ependymoma, GFAP positive
2006 [11]	Mukhida et al.	43	M	Transsphenoidal, gross total resection	Typical ependymoma with rosettes, ring-like and dot-like EMA positivity, GFAP negative. Confirmatory ultrastructure
1956 [13]	Sarkisian and Schultz	31	F	Craniotomy	Typical histology of ependymoma
2001 [14]	Thomson et al.	64	M	Transsphenoidal, partial resection	Typical histology of ependymoma, GFAP positive
2009 [17]	Scheithauer et al.	71	M	Transsphenoidal, subtotal resection	Monomorphous spindle cells with no definite pseudorosettes. Dot-like EMA positivity, cytokeratin CAM 5.2 positive, GFAP negative. Confirmatory ultrastructure
2010 [18]	Belcher et al.	37-65	F	Three time craniotomies and one transsphenoidal surgery	Typical ependymoma with pseudorosettes. GFAP positive, ring-like and dot-like EMA positivity

Ependymomas can develop wherever ependymal cells exist [2]. Sellar ependymomas are hypothesized to originate from the neoplastic transformation of remnants of the ependymal cleft, which consists of undifferentiated ependymal precursors that form the pituitary infundibulum and normally recede by 16 weeks' gestation in the human fetus [10,11]. Sellar ependymomas could develop through a different ependymal cell migration system. Ependymal cells are embryological derivatives of primitive neuroectodermal cells that migrate toward the cortex from periventricular regions. Therefore, ependymal cells are found in parts of the central nervous system at varying distances from the ventricular ependyma. These ectopic ependymal cells might originate the supratentorial extraventricular ependymomas that do not appear to have any ventricular connections, including sellar ependymomas [11,24].

Taylor et al. [25] demonstrated that subgroups of ependymoma either maintain or recapitulate the developmental expression profiles of anatomically restricted progenitor cells, which were identified as radial glial cells (RGCs), and RGCs are likely the origin of ependymoma. RGCs serve as ubiquitous precursors that generate neurons and glia. Ependymal cells, from which ependymomas arise, are derived from RGCs during embryogenesis [26]. Genetic mutations in RGCs may lead to their transformation into cancer stem cells of pediatric ependymomas [27]. Supratentorial ependymomas are characterized by elevated expression of members of the Notch and EphB-Ephrin signaling pathways, and it is likely that overactivation of these pathways may induce neoplastic transformation of RGCs in the cerebral subventricular zone. Since RG-like cells are present not only during development but also persist in the adult central nervous system, especially in the subventricular zone and the spinal cord, RG-like cells may play a role as the origin of adult ependymomas [27,28].

Typically, the immunohistochemical staining pattern of ependymoma is positive for GFAP, vimentin, and S-100 protein [29]. EMA staining frequently appears as punctate, dot-like findings [30]. The majority of ependymomas demonstrate immunoreactivity for cytokeratin AE1/AE3. Electron microscopy can facilitate the diagnosis of ependymoma by showing epithelial differentiation, which includes desmosomal junctions, microvilli, cilia with basal bodies, and intercellular microlumina containing variable numbers of microvilli and cilia [31]. The morphological and immunohistochemical findings supported ependymal differentiation. In the present case, IHC analysis failed to detect IDH1 R132H and TTF-1, and CD34 was not expressed. According to the 2021 WHO classification, molecular testing for gene fusions is required for precise classification [7]. In the present case, molecular analysis using RT-PCR failed to detect any gene fusions; thus, the tumor was interpreted as supratentorial ependymoma, NEC.

The patient exhibited moderate hyperprolactinemia, with a prolactin level of 333 ng/mL on preoperative examination. Immunohistochemical examination showed that the tumor was immunonegative for PRL. We supposed that the hyperprolactinemia was mainly attributable to pituitary stalk compression and oral contraceptive use, given the postoperative decrease in prolactin levels. Concurrently, hypogonadotropic hypogonadism caused amenorrhea both before and after surgery.

In Japan, severe adult growth hormone deficiency (AGHD) is diagnosed based on peak growth hormone (GH) concentrations (<9 ng/mL) during GH provocative tests, such as the GHRP-2 stimulation test [32,33]. GH stimulation tests performed both before surgery (peak, 4.0 ng/mL) and after surgery (2.9 ng/mL) revealed severe AGHD.

The clinical outcome and optimal treatment of ependymomas of the sella turcica are not well established. Armstrong et al. [4] reported that decreased overall survival and worse recurrence-free survival in patients with ependymoma were associated with intracranial location compared with spinal cord location. Gross total resection is regarded as the mainstay of treatment for adult intracranial ependymomas, whereas the role of postoperative radiotherapy after gross total resection remains controversial [34]. In the present case, no adjuvant therapy was administered after gross total resection, and no recurrence has been observed one and a half years after surgery. Longer surveillance is nevertheless required because ependymomas may recur late [34].

## Conclusions

This case report documents an exceptionally rare presentation of a supratentorial ependymoma arising in the suprasellar region, contributing valuable information to the limited worldwide literature on this uncommon entity. The diagnosis was established through a multidisciplinary analysis that incorporated advanced histopathological, immunohistochemical, ultrastructural, and molecular techniques. This case highlights the critical importance of including ependymoma in the differential diagnosis of suprasellar masses, particularly when conventional imaging is atypical for more common lesions such as pituitary adenomas, craniopharyngiomas, or meningiomas. The targeted fusion testing performed in this case did not identify the alterations examined, which does not exclude the diagnosis of ependymoma when classical histopathological, immunohistochemical, and ultrastructural criteria are fulfilled. Because the assay covered a predefined set of fusion transcripts, the molecular classification of this tumor remains incompletely resolved, and morphological assessment retains an important complementary role alongside molecular techniques. While the optimal treatment strategy for sellar ependymomas requires further definition through multicenter collaborative efforts and international case registries, complete surgical resection may be associated with favorable local control in selected cases. However, the optimal management remains uncertain because of the rarity of this entity.
